# Gustatory dysfunction in euthyroid primary hypothyroidism

**DOI:** 10.4314/gmj.v57i4.3

**Published:** 2023-12

**Authors:** Afshan Z Hasan, B L Preethi, Pramila Kalra

**Affiliations:** M. S. Ramaiah Medical College, Bengaluru, Karnataka, India

**Keywords:** Gustatory, Hypogeusia, Primary Hypothyroidism, Euthyroidism, Taste threshold

## Abstract

**Objectives:**

The study assessed gustatory functions in patients with primary hypothyroidism who are euthyroid on supplemental hormone therapy with levothyroxine over six months' duration and to evaluate the association of gustatory dysfunction, if any, with the serum TSH levels.

**Design:**

This analytical community-based cross-sectional study was conducted in April 2021, following participants' ethical approval and written informed consent.

**Setting:**

The study was conducted in a tertiary health care centre in Bangalore, Karnataka, India.

**Participants:**

Sixty-eight subjects participated in this study: 34 primary hypothyroid patients and an equal number of healthy controls.

**Interventions:**

Gustatory sensations were assessed by the triple drop test, and scores were given depending on the identification of the tastants (sweet, sour, salty, and bitter). The taste scores were compared, and the association between TSH levels and gustatory parameters were evaluated.

**Results:**

Overall taste scores were lesser in hypothyroid patients. This finding depicted that their taste thresholds were increased and were statistically significant (p < 0.001), though the association between the degree of hypogeusia and TSH levels was not statistically significant.

**Conclusion:**

Patients with primary hypothyroidism can suffer from hypogeusia, which may revert to normal once they achieve euthyroid status with levothyroxine supplementation. However, this has not been conclusively shown in studies. Our study concluded that hypogeusia was present in primary hypothyroidism despite patients being euthyroid on hormone supplementation, and it was not dependent on the serum TSH levels.

**Funding:**

None declared

## Introduction

Hypothyroidism is a clinical condition due to inadequate thyroid hormone production or inadequate effect on the target tissues. Most cases (95%) are due to primary causes such as idiopathic, surgical, and autoimmune disorders, radioactive iodine ablation therapy, and the thyroid gland's failure to produce adequate thyroid hormones. The thyroid hormone has extensive effects on various body functions. It can affect sensory modalities like taste sensations, which thyroid function changes might impair.^1^

The taste sensation is among the two essential special senses necessary to elicit the flavour of all edible substances, and thus, human beings can relish or reject food. Taste alterations may cause a decline in the quality of life or expose an affected individual to situations that might be hazardous to health, for example, accidental intake of putrified or toxic substances. However, many patients remain unaware of their dysgeusia, which may be subtle and remain unreported to their physicians.

The exact mechanism of hypogeusia in hypothyroidism is not known. Putative mechanisms may be alteration at molecular and chemical levels in the taste receptors or maturation and specialisation of taste buds modulated by the thyroid hormone.^2^, ^3^

Few studies have been conducted to assess gustatory function in primary hypothyroid patients in India, where hypothyroidism is around 11%, compared with only 2% in the UK and 4 6% in the USA.^4^ The present study was done considering a scarcity of data on gustatory dysfunction in primary hypothyroidism.

## Methods

This cross-sectional study was conducted in the Department of Endocrinology in a tertiary care centre between February 20th, 2021 and April 1st, 2021. The study protocol was approved, and ethical clearance (MSRMC/EC/SP-11/02-2021) was obtained from the Institutional Ethics Committee (Reg. No. ECR/215/Inst/KA/2013/RR-19), and written informed consent was obtained from all the participants before commencing the study.

The sample size was determined based on a previous study by Baskoy K et al.^12^ It found that taste function did not differ between sweet, salt and sour tastes, but bitter taste improved after thyroxine supplementation at 6.64 ±0.96 in controls and 6.58 ± 1.2 in patients. In the present study, considering the effect size of 0.729, power of 80% and alpha error of 5 %, the sample size is calculated to be 34 in each group. The cases comprised 34 primary hypothyroid patients aged 18 to 50 years who were euthyroid on treatment with levothyroxine over the last six months. The controls included an equal number of subjects, age- and gender-matched.

Patients with complications like liver and or kidney disease, history of psychiatric or neurodegenerative disorders, diabetes, oropharyngeal trauma/ surgery, olfactory sensation abnormalities, respiratory tract infections, diseases of the oral cavity, using dentures, Vitamin B12 deficiency, pregnancy, breastfeeding females, alcoholics, smokers, pan/ tobacco and other substance abusers, and any subject who had COVID 19 within the past six months, were excluded from the study. The subjects selected in the study were based on the biochemical evidence of normal thyroid functions (TSH, FreeT4). HbA1C was done to rule out prediabetes/ diabetes. Smell sense was tested using coffee beans, peppermint and clove oil. Those who reported hyposmia/ parosmia or anosmia were excluded from the study.

Evaluation of taste sensation was done by using the triple drop test.^5^ Subjects were tested in the morning hours between 9 a.m. and 12 noon, either with an overnight fast or 2 hours after having eaten food. The four basic tastants, namely sweet, salty, sour, and bitter, were presented to the subjects. The concentrations of each tastant used were prepared in 5 dilutions in 50 % steps (Table 1). Samples were randomly given as three drops on the anterior aspect of the extended tongue, swished in the mouth for five seconds, and spit out. The subject was asked to rinse the mouth thoroughly with water between each task. A higher concentration of tastant was presented if the subject could not identify the taste.

**Table 1:** Concentrations of taste stimuli used (G/Ml) and the allotted scores

Dilution Steps	Score	Sucrose	Citric Acid	Sodium Chloride	Quinine Hydrochloride
**1**	1	3	2.5	1	0.02
**2**	2	1.5	1.2	0.5	0.01
**3**	3	0.75	0.6	0.25	0.005
**4**	4	0.38	0.3	0.12	0.0025
**5**	5	0.19	0.15	0.06	0.0012

Scoring depended on identifying concentrations from “1” to “5”, 1 being the most concentrated and 5 being the least concentrated solution for the particular tastant. The degree of hypogeusia in primary hypothyroidism was also compared with serum TSH values. Cases were divided into two groups: those with a serum TSH level of <3.0 mU/L and those with a serum TSH ≥3.0 mU/L, as depicted in Table 3. All these patients were euthyroid (serum TSH levels of 0.4 to 4.0 mU/L according to the American Thyroid Association.^6^)

**Table 3:** A Comparison of the mean and standard deviation of the different taste scores

Variables	Cases	Controls	Total	P Value
**Sweet**	3.21±0.84	4.71±0.52	3.96±1.03	<0.001**
**Sour**	4.53±0.56	4.97±0.17	4.75±0.47	<0.001**
**Salty**	4.15±0.7	4.76±0.43	4.46±0.66	<0.001**
**Bitter**	2.74±1.11	4.32±0.81	3.53±1.25	<0.001**

### Statistical Analysis

Statistical analyses were performed using the Statistical Package for the Social Sciences 21 for Windows (SPSS Inc., Armonk, NY, USA), and p-values ≤0.05 were considered significant. The quantitative variables were analysed using descriptive statistics such as mean and standard deviation. The qualitative variables were analysed using frequency and percentage. Student t-test was used to test the difference in the mean values between the two groups.

## Results

The present study was done to assess gustatory functions in primary hypothyroidism, i.e., whether the taste thresholds of the four tastants (sweet, sour, salty, and bitter) vary amongst the primary hypothyroid cases who are euthyroid on treatment, as compared to the controls, showed that taste scores for all the tastants were lesser in patients as compared to controls (Tables 2 and Table 3). The results implied that the taste thresholds of the four tastants were increased in cases (p-value < 0.001). The bitter taste threshold was most affected, followed by sweet, salty, and sour.

**Table 2:** Taste scores distribution amongst cases and controls

Taste Scores	Cases	Controls	Subjects
	**N=34**	**N=34**	**Total= 68**
**Sweet**			
**1**	1(2.9%)	0(0%)	1(1.5%)
**2**	5(14.7%)	0(0%)	5(7.4%)
**3**	15(44.1%)	1(2.9%)	16(23.5%)
**4**	12(35.3%)	8(23.5%)	20(29.4%)
**5**	1(2.9%)	25(73.5%)	26(38.2%)
**Sour**			
**1**	0(0%)	0(0%)	0(0%)
**2**	0(0%)	0(0%)	0(0%)
**3**	1(2.9%)	0(0%)	1(1.5%)
**4**	14(41.2%)	1(2.9%)	15(22.1%)
**5**	19(55.9%)	33(97.1%)	52(76.5%)
**Salty**			
**1**	0(0%)	0(0%)	0(0%)
**2**	0(0%)	0(0%)	0(0%)
**3**	6(17.6%)	0(0%)	6(8.8%)
**4**	17(50%)	8(23.5%)	25(36.8%)
**5**	11(32.4%)	26(76.5%)	37(54.4%)
**Bitter**			
**1**	4(11.8%)	0(0%)	4(5.9%)
**2**	12(35.3%)	0(0%)	12(17.6%)
**3**	9(26.5%)	7(20.6%)	16(23.5%)
**4**	7(20.6%)	9(26.5%)	16(23.5%)
**5**	2(5.9%)	18(52.9%)	20(29.4%)

Hypogeusia in primary hypothyroidism was further evaluated for association with serum TSH levels within the euthyroid range. Of the 34 cases of primary hypothyroidism under study, 22 had TSH<3.0 m U/L, and 12 had TSH ≥3.0 m U/L. Taste scores of both these groups were evaluated (Table 4). This was not found to be statistically significant.

**Table 4:** TSH- frequency distribution of taste scores of cases studied

Taste cores of cases	TSH <3.0(N=22)	TSH ≥3.0(N=12)
**Sweet**		
**1**	1(4.5%)	0(0%)
**2**	3(13.6%)	2(16.7%)
**3**	11(50%)	4(33.3%)
**4**	7(31.8%)	5(41.7%)
**5**	0(0%)	1(8.3%)
**Sour**		
**1**	0(0%)	0(0%)
**2**	0(0%)	0(0%)
**3**	1(4.5%)	0(0%)
**4**	11(50%)	3(25%)
**5**	10(45.5%)	9(75%)
**Salty**		
**1**	0(0%)	0(0%)
**2**	0(0%)	0(0%)
**3**	5(22.7%)	1(8.3%)
**4**	11(50%)	6(50%)
**5**	6(27.3%)	5(41.7%)
**Bitter**		
**1**	3(13.6%)	1(8.3%)
**2**	9(40.9%)	3(25%)
**3**	7(31.8%)	2(16.7%)
**4**	2(9.1%)	5(41.7%)
**5**	1(4.5%)	1(8.3%)
**Taste cores of cases**	TSH <3.0 (N=22)	TSH >3.0 (N=12)
**Sweet**		
**1**	1(4.5%)	0(0%)
**2**	3(13.6%)	2(16.7%)
**3**	11(50%)	4(33.3%)
**4**	7(31.8%)	5(41.7%)
**5**	0(0%)	1(8.3%)
**Sour**		
**1**	0(0%)	0(0%)
**2**	0(0%)	0(0%)
**3**	1(4.5%)	0(0%)
**4**	11(50%)	3(25%)
**5**	10(45.5%)	9(75%)
**Salty**		
**1**	0(0%)	0(0%)
**2**	0(0%)	0(0%)
**3**	5(22.7%)	1(8.3%)
**4**	11(50%)	6(50%)
**5**	6(27.3%)	5(41.7%)
**Bitter**		
**1**	3(13.6%)	1(8.3%)
**2**	9(40.9%)	3(25%)
**3**	7(31.8%)	2(16.7%)
**4**	2(9.1%)	5(41.7%)
**5**	1(4.5%)	1(8.3%)

Thus, the serum TSH levels, either towards the lower limit or the upper limit of the normal range, do not significantly influence the degree of hypogeusia in primary hypothyroidism.

## Discussion

Taste sensation gets altered in several disorders like endocrine disorders, nutritional disorders, diabetes mellitus, and chronic liver and kidney diseases.^7^,^8^ Physiological alterations in the internal signal transduction pathways can modulate gustatory preferences.^9^ Serum sodium and blood glucose levels also affect taste sensations.^10^ Such observations lead to the speculation that gustatory responses may change in endocrine disorders like hypothyroidism, which causes many metabolic alterations. Loss of appetite is commonly seen as a presenting symptom of hypothyroidism. In a study by McConnel et al., eighteen hypothyroid patients' smell and taste functions were tested. Dysgeusia was observed in 50% of the patients, with bitter taste hampered the most. The study concluded that both smell and taste deficits are observed in hypothyroidism, which treatment could reverse. Further, a taste defect could explain appetite alterations and may have indirectly resulted from changes in body weight and blood sugar fluctuations.^11^

This study also concluded that taste sensations are decreased in primary hypothyroidism patients even when they were euthyroid on treatment, of which bitter taste sensation is affected the most.

Baskoy et al. found that dysgeusia in hypothyroid patients improved after treatment with levothyroxine.^12^ Primary hypothyroid subjects in our study were euthyroid on levothyroxine replacement for at least six months but still had hypogeusia. Contrary to our findings, a study conducted by Deniz et al. concluded that newly diagnosed primary hypothyroid hypogeusia patients show an improvement in taste sensation after thyroid hormone replacement therapy of 3 to 6 months duration.^13^

A study on experimentally rendered hypothyroid rats indicated that these animals consume higher salt solutions than hyperthyroid rats.^14^ In yet another study, hypothyroid rats demonstrated a preference for saline.^15^ The answer to these observations could be explained based on a study by Taylor and Fregley, which concluded that hypothyroid rats are less responsive to administered aldosterone.^16^

Therefore, the present study aimed to better evaluate the taste changes in primary hypothyroidism with euthyroid patients for at least six months on continued levothyroxine supplementation therapy. Alterations in gustatory responses were present in these subjects despite maintaining a euthyroid state. Furthermore, the taste thresholds for all four tastants, sweet, salty, sour, and bitter, were increased compared to the controls. This shows that hypothyroid subjects could recognise a higher concentration of these tastants.

However, the exact pathogenesis of alterations in taste sensations in primary hypothyroid patients is unknown. Thyroid hormones play an important role in the maturation and specialisation of taste buds. Taste receptor cells release neurotransmitters on excitation, just like neurons.^2^ The origin of taste cells is epithelial, unlike ectodermal neurons.^17^ There is continual regeneration of taste cells throughout life due to mechanical and molecular mechanisms.^18^ A study done by Law JS et al. concluded that thyroid hormone inhibits purified taste bud membrane adenosine 3′,5′-monophosphate phosphodiesterase activity. This increases intracellular cAMP within the taste buds, modulating the action of thyroid hormone in the tasting process.^3^

T3 levels were not done as part of the routine investigations for the subjects in this study. TSH and free T4 were tested in this study, and TSH follow-ups were done. Subsequent research can be done to find out whether T3 supplementation can help restore taste sensations in hypogeusics. Lowering TSH values still further within the normal limits might also alleviate hypogeusia. Further studies with larger sample sizes are suggested to elaborate on why hypogeusia persists in primary hypothyroidism despite adequate treatment and maintaining the euthyroid state. Studies can also be carried out to know the specific cause of hypogeusia in primary hypothyroidism.

## Conclusion

The subjects exhibit hypogeusia despite maintaining a euthyroid state with medical supplementation. The degree of hypogeusia does not change with varying serum levels of TSH amongst the euthyroid subjects.
